# Challenges of being a maternity service leader during the COVID-19 pandemic: a descriptive analysis of the journey

**DOI:** 10.1186/s12884-023-05614-5

**Published:** 2023-04-24

**Authors:** Annie Tan, Alyce N. Wilson, Tracey Bucknall, Robin Digby, Joshua P. Vogel, Caroline SE. Homer

**Affiliations:** 1 Maternal, Child and Adolescent Health Program, Burnet Institute, Melbourne, Australia; 2 School of Nursing and Midwifery, Deakin University, Melbourne, Australia; 3 Centre for Quality and Patient Safety Research-Alfred Health Partnership, Alfred Health, Melbourne, Australia

**Keywords:** Maternity care, Leader, Policy, Director, Unit coordinator, Manager, Obstetrician, Midwife, Midwifery unit manager, COVID-19, Pandemic, Longitudinal qualitative study, Challenges, Communication, Information, Adapting, Support, Response

## Abstract

**Background:**

In Australia, maternity care services provide care for pregnant and postpartum women and their newborns. The COVID-19 pandemic forced these services to quickly adapt and develop policies and procedures for dealing with transmission in health care facilities, as well as work under public health measures to counter its spread within the community. Despite well-documented responses and adaptations by healthcare systems, no studies have examined the experiences of maternity service leaders through the pandemic. This study aimed to explore the experiences of maternity service leaders, to understand their perspectives on what happened in health services and what was required of a leader during the COVID-19 pandemic in one Australian state.

**Methods:**

A longitudinal qualitative study collected data from 11 maternity care leaders during the pandemic in the state of Victoria. Leaders participated in a series of interviews over the 16-month study period, with a total of 57 interviews conducted. An inductive approach to developing codes allowed for semantic coding of the data, then a thematic analysis was conducted to explore patterned meaning across the dataset.

**Results:**

One overarching theme, ‘challenges of being a maternity service leader during the pandemic’, encompassed participant’s experiences. Four sub-themes described the experiences of these leaders: (1) needing to be a rapid decision-maker, (2) needing to adapt and alter services, (3) needing to filter and translate information, and (4) the need to support people. At the beginning of the pandemic, the challenges were most acute with slow guideline development, rapid communications from the government and an urgent need to keep patients and staff safe. Over time, with knowledge and experience, leaders were able to quickly adjust and respond to policy change.

**Conclusion:**

Maternity service leaders played an important role in preparing and adapting services in accordance with government directives and guidelines while also developing strategies tailored to their own health service requirements. These experiences will be invaluable in designing high quality and responsive systems for maternity care in future crises.

**Supplementary Information:**

The online version contains supplementary material available at 10.1186/s12884-023-05614-5.

## Background

The COVID-19 pandemic challenged health services across the globe. In every country, governments, policy makers and healthcare leaders rapidly reallocated resources, redeployed staff to prevention and treatment activities, and dedicated wards or constructed entire hospitals to treat COVID-19 patients [1, 2]. Public health measures to curb transmission such as international travel bans, mandatory quarantine for returning travellers including staying in government controlled centres for up to two weeks, minimised movement in the community, and stay at home orders known as lockdowns were some measures employed [3–5]. At an individual level, governments introduced social or physical distancing, promoted good hand hygiene practices and introduced mandatory face mask in enclosed areas [6, 7]. Australia, in the global context, initially saw fewer confirmed COVID-19 cases or deaths compared with similar countries [8]. However, in the first year of the pandemic, the state of Victoria had more than 20,000 cumulative confirmed cases, four times greater than the most populous state of New South Wales, and significantly higher than all other states and territories [9]. Breaches in mandatory quarantine facilities, virus clusters in essential workplaces, and a complacent younger demographic were some of the reported reasons for increased COVID-19 cases in Victoria [10, 11]. The response in Victoria therefore was considerably stricter than across the rest of Australia including two long lockdown periods (16–20 weeks in 2020) and two shorter lockdown periods (2–4 weeks) by July 2021, a nightly curfew, 5 km radius limits from home to restrict movement and work from home orders for all non-essential workers [12, 13].

Changes to policy and practice within the Victorian health system occurred immediately as part of the response and to manage the surging COVID-19 cases and hospital admissions [14]. All aspects of the health system were affected either directly, through infections and illnesses, or indirectly, as healthcare workers were reallocated to frontline services, routine medical procedures and non-urgent health services were cancelled or postponed (e.g. elective surgeries), and health services reduced face-to-face appointments and transitioned to telehealth services [15, 16].

The maternity care service system continued to operate throughout the pandemic. However, adaptations were required, including preparing for COVID-19 positive pregnant and postpartum women [17–19]. Changes to maternity service policy and practice were frequent, especially in birthing suites, and restrictions to the presence of support people during antenatal appointments, labour and birth and in the postpartum period varied significantly [20, 21]. The uncertainty and unknown impact of COVID-19 required healthcare workers to remain vigilant to nosocomial infection, transmission, and disease burden with personal protective equipment (PPE) becoming common parlance, and many staff worked from home where possible [22, 23]. The public health orders, especially during the first year, were constantly changing and all health services were in a constant state of disruption and uncertainty.

The provision of effective and compassionate management and leadership through the first 12–16 months of the pandemic in Victoria was critical. More than 75,000 women gave birth in 2020 in Victoria and every one of them was impacted in some way by the constantly changing public health system [24]. Limited supports and sudden cancellations to appointments in the early adjustment period, negatively impacted women’s experiences [25]. To ensure women continued to receive high quality care, there is an explicit need for leaders in maternity services to adapt and respond to sudden changes like a public health crisis accordingly. The leaders and managers directing their care played a key part in the COVID-19 response, although they have remained mostly invisible in a pandemic that focused on infectious disease management, and critical or intensive care. This study aimed to explore the experiences of maternity care leaders, to understand what happened from their perspectives and recognise what was required of a leader during the COVID-19 pandemic in one Australian state.

## Method

### Design

We used a longitudinal qualitative study. Longitudinal qualitative research (LQR) is distinguished from other qualitative approaches by the way in which time is designed into the research process, making change a key focus for analysis [26]. Duration, time and change are key principles that underpin LQR, recognising that time and change are contextual and may transform over the course of a study [27]. This study follows one of Holland’s methodological models of LQR; in which an original study of participants are followed up over a period of time [28]. Given the rapid changes to maternity care and health service delivery, this design was appropriate to explore the experiences of the maternity service leaders over 16 months of the COVID-19 pandemic. The Consolidated criteria for reporting qualitative research (COREQ) checklist (Appendix 1) was also used to ensure explicit and comprehensive reporting of qualitative studies [29]. Maternity service leaders will be referred to as ‘leaders’ throughout the study.

The research team included a diverse group of nursing and midwifery researchers and public health professionals with extensive experience in qualitative research. The team consisted of five females and one male, all of which have had experience in patient-facing roles within maternal and child health. Their knowledge and expertise in health service delivery allow a focused conceptualisation of the project, as well as deep interpretation and understanding of the data.

### Setting

This study took place in Victoria, a state that had significantly higher numbers of COVID-19 cases in 2020 than in other parts of Australia. A “State of Emergency” was declared on the 16th March 2020 [30] and the Victorian government then pursued an aggressive suppression strategy to minimise the transmission of the virus. This included imposing lockdowns, that is, restricting movement of the public, and a consistent message to “Stay at home” [31]. Lockdowns were categorised in four stages: Stage 1 imposed restrictions on social gatherings indoors and outdoors; Stage 2 included restrictions on non-essential venues and activities; Stage 3 was restrictions on movement, with only five reasons to leave home (to attend work, shop for necessary items, to study, to provide or receive care, or for exercise) with further additional rules added throughout Stage 3; and, finally, Stage 4, in which additional restrictions were implemented, limiting movement throughout metropolitan areas, permitting only travel within 5 km (kms) of the home, curfews and mandatory face masks. Metropolitan Melbourne, the geographical area defining Melbourne as a city and the capital of the state of Victoria, is home to more than 70% of Victorian population [32]. In total, Melburnians have endured six lockdowns since the start of the pandemic. Four of these occurred within the study period.

There are 53 maternity units across the state of Victoria, including 16 private facilities. Pregnancy and birth care options for women in Victoria vary according to geographical location, models of care available in the area, and past medical history [33]. Women have the option to receive care as a public or private patient and are often referred to antenatal clinics by their local general practitioner. The maternity system is set up to provide care in 6 levels: Levels 1–3 provide local care for healthy women and babies at low risk; Level 4 provides local care for women and babies with some risk requiring additional care; and Levels 5–6, which provide local care for all women and babies, regional and state wide care for women and babies with complex pregnancies and births requiring neonatal intensive care [34]. In 2020, a total of 75,870 women gave birth to 76,990 babies [35].

### Sample and recruitment strategy

Midwifery, obstetric and neonatal leaders, and policy makers providing maternity services across Victoria were invited to participate in this study very early in the pandemic. Purposive sampling was undertaken using networks within Safer Care Victoria (SCV) an administrative department of the Department of Health, Consultative Council on Obstetric and Paediatric Mortality and Morbidity (CCOPMM) an independent body in Victoria that reviews cases of maternal, perinatal and paediatric mortality and morbidity, and the Victorian health system. These leaders were invited to participate as they were playing, or were likely to play, a crucial role in maternal and newborn health policy development and implementation during the pandemic. The aim was to invite leaders from different levels or types of services. Invitations were issued via email in late March 2020. In total, 13 participants were invited to participate in the study, one invited person did not respond and another was excluded after the initial interview as their experience was outside the maternity care sector. Eleven participants were included in the study, with one male participant broadly representing the gender mix in maternity care in the state (Table 1).

### Data collection

Interviews were conducted from late March 2020 to the end of July 2021, by CSEH, a highly experienced qualitative health researcher with a midwifery background. For the final interviews, AT was invited to participate between June-July 2021 with verbal consent being obtained from all participants prior to the scheduled interview date. She observed interview techniques and tool field notes from discussions.

Interviews were conducted online using the Zoom platform. Most interviews were short (20–30 min) and conducted at different time points depending on availability of participants and the circumstances of the pandemic (what was happening in terms of changes or stability). The interviewer was able to establish a relationship and build a rapport with participants, obtaining their trust and commitment to the study. Contact was made via email inviting participants for another interview. The number of interviews per leader was highly dependent on their availability and work commitments, which resulted in three to eight interviews per participant (Table 1).

**Fig. 1:**
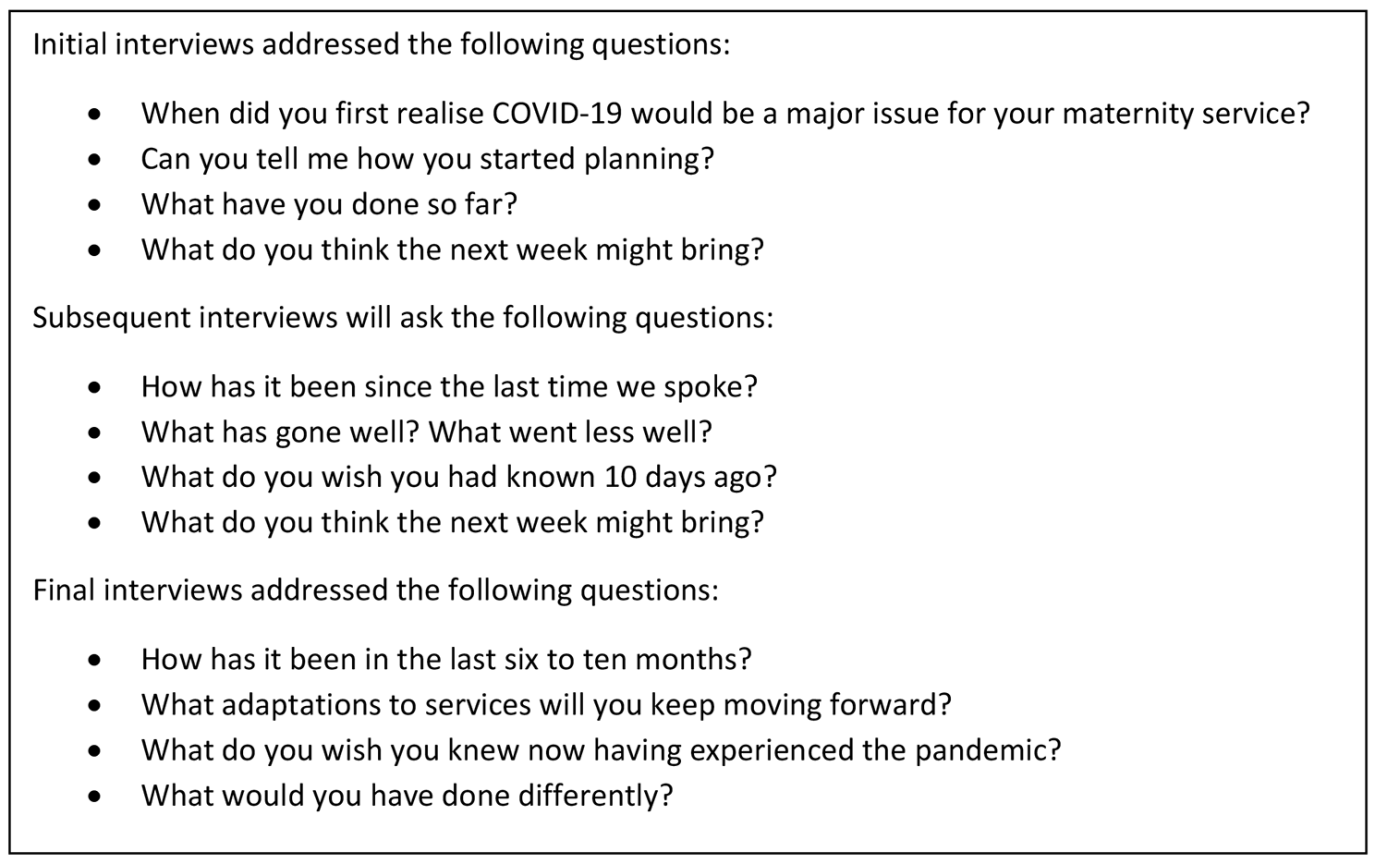
Sample of questions addressed by participants in interviews throughout the study period

An interview guide (Fig. 1) was designed to encourage participants to provide broad and general descriptions of their institution’s COVID-19 response. The same questions were asked throughout all interviews to elicit what happened, what could have been done differently and what they anticipated would occur in the coming weeks. Whilst acknowledging the repetitive nature of the questions, participants’ familiarity with what questions to expect during the interviews allowed them to reflect on the time that had passed and provide rich encounters with each interview. Field notes were taken during interviews, enabling the interviewer to recap what was previously discussed.

### Analytical strategy

Interviews were transcribed verbatim and imported into QSR NVivo 20 coding software for analysis using Clarke and Braun’s six phases of thematic analysis [36]: data familiarisation, data coding, initial theme generation, theme development and review, theme refining, defining and naming and writing up [36]. One researcher (AT) deeply immersed herself in the data through reading and re-reading transcripts and listening to the audio recordings. An inductive orientation to coding the data was utilised, obtaining semantic codes to determine explicitly shared ideas, views and experiences of participants [37]. Manual codes were developed using relevant quotes and anecdotes and were grouped in NVivo 20 coding software according to relevancy. Quotes relevant to the research aim were edited to remove colloquial mannerisms using ellipses and where additional information was required square brackets were used. Approximately eight transcripts (14%) were reviewed by a second author (AW) to ensure that there was a consensus with the consistency of coding of data. Both coders are healthcare professionals with experience in patient-facing roles in maternity hospitals, (AT and AW) worked clinically in the maternity sector throughout the pandemic, and AW also brought experience in public health medicine. Both researchers had no prior relationship with participants, and transcripts were de-identified to preserve anonymity and reduce any biases. Initial theme generation was conducted by two members of the team and codes were analysed to explore patterned meaning across the coded data. Provisional themes were then discussed with broader research team to refine, define and name themes.

Final manuscript drafts were sent to all participants for comments and review, to ensure that authors successfully de-identified leaders’ roles and their healthcare setting. Themes were also reviewed and all the participants indicated they felt the analysis reflected their broad experiences.

## Results

Eleven participants were interviewed (2 senior executives, 3 obstetricians, 2 midwives, 1 maternity unit manager, 1 neonatal unit manager, 1 neonatologist, 1 GP obstetrician). These leaders worked in diverse clinical settings across Victoria, within SCV, CCOPMM, metropolitan tertiary hospitals, regional tertiary hospitals, private practices, or a combination of these (Table 1).

**Table 1:** Breakdown of participant’s roles within maternity care and the number of interviews they participated in

Professional classification	Role	Local (metropolitan or regional)	Access level (public or private)	Health service level	Number of interviews conducted	
Midwife	Senior executive (policy)	Both	Public	Responsible for all levels	6	
Medical	OBGYN*	Metropolitan	Public	Level 6	8	
Medical	OBGYN*	Both	Public	Level 5	6	
Medical	GP** Obstetrician	Both	Regional	Level 5	6	
Midwife	Consultant midwife	Metropolitan	Public	Level 6	6	
Midwife	Consultant midwife	Metropolitan and regional	Public	Level 4 & 6	5	
Midwife	Maternity unit manager	Metropolitan	Public	Level 6	5	
Medical	Senior executive (director)	Regional	Public and private	Level 4	4	
Medical	Paediatrician	Metropolitan	Public	Level 6	3	
Medical	OBGYN*	Metropolitan	Public	Level 6	4	
Nurse	Neonatal unit Manager	Metropolitan	Public	Level 6	4	
**TOTAL**					**57 interviews**	

*OBGYN refers to obstetrician and gynaecologist

** GP refers to general practitioner

**Fig. 2:**
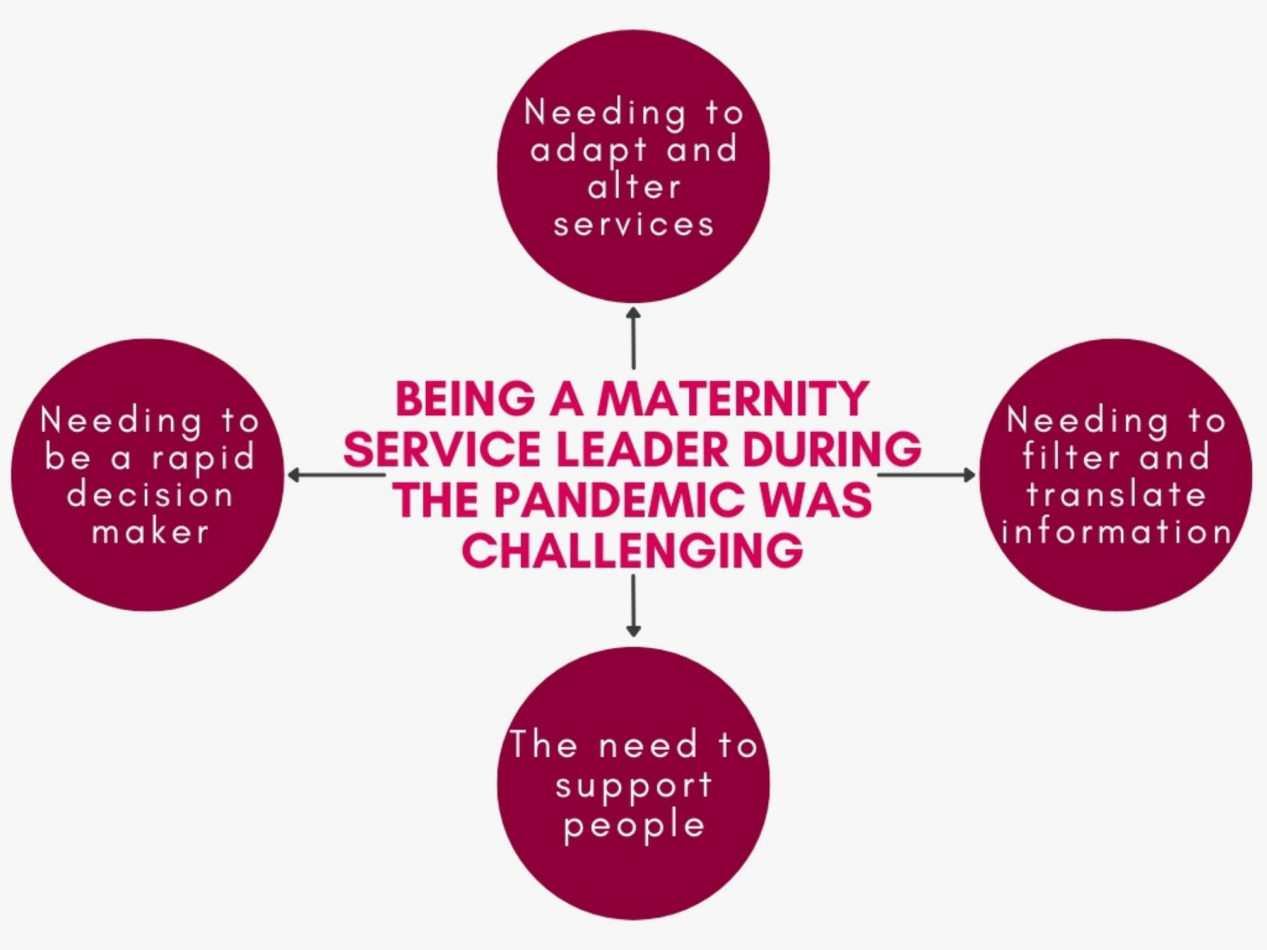
Infographic that describes the over-arching theme in the middle, and sub-themes surrounding

The overarching theme ‘being a maternity service leader during the pandemic was challenging’ encompassed participant’s experiences. Four sub-themes described the experiences of these leaders: (1) needing to be a rapid decision-maker, (2) needing to adapt and alter services, (3) needing to filter and translate information, and (4) the need to support people (Fig. 2).

### Being a maternity service leader during the pandemic was challenging

The overarching theme and challenges faced by maternity care leaders resonated strongly throughout the interviews. With little initially known about the SARS-CoV-2 virus and its impact on pregnant women and newborns, leaders were under pressure to quickly respond despite the uncertainties and ensure that quality care could continue to be provided, as explained here:

“I think as a clinician, I know that one of the hardest—and again this comes back to uncertainty and change—one of the hardest things as a clinician is to sit in uncertainty and to be able to say to someone, “I don’t know … I don’t know what’s going to happen” (Medical leader A).

Despite working in different health services, the leaders faced similar challenges, including the need to rapidly adapt and develop specific and tailored guidelines to respond to COVID-19 while managing and supporting the workforce through unprecedented times. Many were working from home, at least some of the time, and they described the lack of social connections and interconnectedness with staff as one of the biggest challenges, as one participant explained, “I feel a bit disconnected as a group, so we are trying to reconnect, because it can be a lonely job as a unit manager and you don’t have your other peers to catch up with that we would do most days …. you feel a little bit isolated, even though you are at work” (Unit manager A). They struggled with the rapid shift in care giving: “in maternity, we want to have that connection, we want to be able to talk and, you know, touch and all of those things and not being able to do that is a very horrible feeling” (Medical leader C).

The sub-themes described below detail challenges that were experienced, exploring what happened as they needed to make rapid decisions, adapt and shift services, filter and translate information and support people, often all at the same time.

#### Needing to be a rapid decision maker

The first 6 months of the pandemic required rapid and critical decision making by those in leadership roles. Leaders of maternity services needed to translate decisions made at the federal and state government level into policy and practice often very quickly. Many described struggling with ever-changing decision making, frequently without warning “we’ve been madly trying to get our heads around it. Most people have got some plans in place now at the hospital and my practice and it’s … what is going to happen now?” (Medical leader B).

Developing COVID-19 response guidelines for their health facilities was a key priority, and leaders initially worked with other departments and colleagues to do so. For example, one participant initially said, “what I saw was people collaborating straight away, being really supportive of each other, personally, professionally and I think that also helped some of the units [who] had started having the conversations but hadn’t yet put anything on paper” (Medical leader D). This leader described how this collaborative approach to guideline development continued and reported that increased uniformity assisted this rapid decision-making process, “we’ve found it to be relatively useful to have the [state government] group to come together and generate guidance, albeit often based more on opinion than hard scientific facts, but it’s something at least” (Medical leader D). However, obtaining evidence to inform these rapid decisions was not always easy: “I think it would have been helpful to have some more guidance, we basically focused mainly on what the Department [of Health] told us, but then … people were quoting the Australian obstetric college, and they were quoting the United Kingdom obstetric colleague, you know you get so many different buy-ins that in the end of the day, I felt that that was the most frustrating part” (Unit manager A). This also changed over time as new guidance was released and new organisations were established and started providing guidance over the first 16 months. Leaders also had to ensure that these guidelines could be easily understood by staff. One leader described taking lengthy guidance from obstetric colleges to create flowcharts: “… the people at the bedside are saying the flowcharts are what they’re really interested in, something simple that they can follow” (Midwifery leader C).

Once developed, the guidelines needed to be implemented as quickly as possible, which created constant disruption for their staff. One leader explained: “I did feel like our brains were going a million miles an hour and we were a small group making all these changes and plans, but because every day the guidance from the government kept changing. We felt that we couldn’t continuously update the staff, it makes it very confusing for them, because it was already confusing for us… we are very mindful not to send too many things out at once. But it always seemed to come to Friday afternoons that we send out memos with changes and that you have to inform staff over the weekend separately, so they are knowing of what they need to do” (Medical leader E). Over the 12 months, guidance kept changing and again this required rapid decision making – what to change and what to keep was a common issue raised. This unit manager described the initial frustrations in April 2020, “I found the hardest probably was the organisation wanted to come up with a plan, you know and you want it to be air tight before you kind of release it to the staff, so that’s probably what the staff have struggled with the most is the ever changing information, because now we are expecting them to adapt on a you know daily shift basis” (Unit Manager A). By June 2020, the unit manager said, “when I spoke to you last we were in kind of that you know in between phase of “do we release information even though it’s ever changing, all the guidelines” and I did get frustrated with that, that my staff didn’t feel like they knew what was going on, but I feel like we waited enough time. We now haven’t needed to change the guidelines, it’s just been add-ons if needed and staff I think feel really informed and feel like we as a whole service of you know [have] provided them with their options in regards to wearing PPE” (Unit Manager A).

Additionally, rapid workforce decisions needed to be made due to the increasing community transmission in Victoria. There was a higher chance of staff being furloughed or required to self-isolate, which demanded additional forward planning on staff arrangements, with its own set of challenges: “we are revisiting all the surge plans, workforce plans and just trying to figure out how we can limit experiencing positive staff members and then having to do the close contact tracing…” (Unit manager A). Again, the guidance and rules around the workforce did not stay the same over the first 16 months providing another example of a constantly moving context. A strong initial response to workforce planning was described by this leader, “we’ve done a lot of work in the background on workforce planning. We have had a lot of staff who have taken up the offer of increasing hours because their financial situation has changed at home, so for the first time in a very long time we’re not using bank or agency staff because we have our own staff, we’re close to full FT because we’ve got our own staff that have taken up the offer of full-time or close to full-time work. From activity perspective we’ll see a bump, but I don’t know about acuity” (Unit manager B). Demonstrating participants reflection on staffing experiences, in a subsequent interview this same leader stated “lockdown if you want to call 4.0 that we just had now was probably the one actually that hit a lot of people harder than lockdown last year. They didn’t understand … I think it was a lot around messaging that was coming out from the government and they didn’t understand… I think there was a lot more frustration from a staffing perspective this time around as well, because I think you are starting to see, I think everywhere now the cracks and people are getting frustrated with the differences between different states and what we are doing and different health services and what we are doing…”(Unit manager B).

#### Needing to adapt and alter services

To accommodate public health measures and physical and social distancing, infrastructure changes were implemented in antenatal clinics, outpatient appointments as well as birthing suites. The management of patient flow required new triage tools for assessing COVID-19 infection risk, as well as reducing the number of patients in waiting rooms. Birthing suites were also re-structured to meet the needs of COVID-19 positive patients and staff caring for these women to ensure there was minimal opportunity for transmission. Many of the leaders were making such service adaptations for the first time, and innovative strategies were implemented to accomplish them: “we have also moved to trying not to have women in the waiting room, so they will come and check in at the desk and then be told they can either go back to their car and wait and we text them when we are ready for the appointment and ask them to come back to clinic to be seen” (Midwifery leader A).

All leaders described shifting from face-to-face consultations to online, telehealth or remote care services, or a combination, as a major adaption. The shift to telehealth was welcomed as a necessary change: “we’ve been waiting over a decade for telehealth and I think that that’s been one of the biggest benefits of the COVID-19 pandemic … that’s probably the biggest change … I think it will be very beneficial moving forward” (Unit manager B). This transition was also supported by this leader’s workforce: “the midwives were describing that they felt they could have a quality conversation, because the phone wasn’t ringing, the door wasn’t opening and shutting, there wasn’t noise outside, they were able to totally focus on the needs of that woman and what that woman was asking in the discussion between them … the midwives are saying that they actually feel it’s a better quality discussion than what might have happened in pre-COVID” (Midwifery leader C).

Shifting to telehealth services did raise some concerns in this leaders practice. At the beginning of the transition, this leader reflected on adapting to telehealth by saying, “I think it’s really hard … it’s quite easy for me, because I know my patients very well, and I’ve been doing this for a long time but it’s difficult for our registrar (specialist-in-training). We have a fantastic registrar, but she’s only been in general practice since February, so she doesn’t know her patients and you have to have a certain amount of confidence and experience I think to conduct telehealth safely” (Medical leader B). It was welcomed as an alternative to face-to-face care, yet at the final interview, this leader said, “I know telehealth and telephone consults have a place, but in antenatal care, it is fraught with difficulty. You really do need to eyeball the patient and have that connection with them and do some basic examinations” (Medical leader B). Rapport building, ability to conduct thorough assessments as well as privacy were other concerns leaders had.

A significant challenge for leaders involved changing support people and visitor policies to align with the broader public health response to COVID-19. During peak infection rates, there were no visitors and support people were not allowed to attend appointments. Many staff reported that whilst women were initially upset with these changes, they were ultimately accepting, acknowledging that these policies were there to protect them and their babies. One leader said: “it’s amazing how the community has respected what the hospitals want and almost respecting us as a profession as well … hopefully this continues and they continue to respect us in our profession, but I think when we now say something, “we are doing this for the safety and wellbeing of you and your family” that they’re actually listening to that and respecting that decision” (Medical leader E).

Throughout interviews there were periods of intense lockdowns, high community transmissions and surges in confirmed COVID-19 cases admitted to hospitals. One leader described the experience as “I think … I just feel like we are very well prepared, well we think we are, so we are kind of waiting for the red women [women with COVID-19] to come in, but I think it will see changes in our workforce and our shifts especially” (Unit manager A). Ultimately, few pregnant and postpartum COVID-19 positive women required hospital admissions at this time in the pandemic despite all the preparatory efforts: “we are now at the point where we are pretty much ready, but we have nothing there and so it’s this very unusual almost limbo and I think “limbo” is the right word of, we are all ready but there are a lot of people sitting around not doing very much now, because we were ready for a surge and ready for something more” (Medical leader A).

Over the first 16 months, service adaptations were constant and leaders described having to keep up; policies and protocols seemed to change by the day: “they’ve changed their minds again and they are bringing back surgery and depending on who you are within the organisation, your head is spinning, because every time you turn around something else is changing and so you know, the two big features of the pandemic and what everybody in the world is experiencing, no matter whether or not they are in health care or politics or journalism or at home with kids or whatever it is, is uncertainty and change and the pandemic has brought both in spades and so in the hospital environment where we are getting asked to make changes constantly and then get ready for the change, make the change and then “okay, now we’re changing again”, very challenging” (Medical leader A). One leader reflected on the impact this had on staff, “they are saying what they’re sick of is the rules changing so many times and they say that has been exhausting and that things are trying to change from us, you know, weekly or daily basis” (Midwifery leader C).

#### Needing to filter and translate information

A key role for leaders was filtering and translating information from government policy updates and communications briefs, and service executives, into lived realities for staff and patients. Leaders were cognisant that information needed to be understandable and practical even through it was often changing. As one leader explained, “it’s been about making sure that the staff have the information they need because they are being inundated and the messaging within [one health service] is very good and we are often ahead of the messaging that comes out from the Department [of Health], so it’s been about filtering that. That takes a bit of time and a bit of getting used to and figuring out the best means of communication because at the end of the day communication is key. … A lot of people have email fatigue at the moment and I think that’s hard when that’s the expectation and how you’re going to get your information out to your staff.” (Unit manager B).

Effective communication was crucial and described by another leader as, “absolutely key to be able to communicate with the sector in a really open and transparent way, I think is really important now [early in the pandemic], but it is going to become a lot more important as things get more difficult” (Midwifery leader B). In the early months, there was the lack of clarity, guidance and clear information for healthcare providers which caused distrust amongst front-line staff and hospital executives. At times, little information was provided to these leaders about the current situation at a state government level which meant that this could not be relayed to staff. One leader said, “I think there was a fair bit of anger I think when things weren’t being communicated well and there was a delay in communication” (Medical leader B). Many leaders explained that they watched the state Premier’s daily press conference to understand what the guidance of the day was going to be and this was a difficult way to be receive information and continually respond.

Additionally, there was no clear and consistent message from a single source, making it difficult for leaders to provide succinct messages to staff. In some cases, leaders reported that their staff felt unhappy, frustrated or disappointed by the lack of information from an executive level. “There are some other things that are not happening that are a major problem and actually I would say that hospital morale is incredibly low at the moment and people are very confused and very frustrated … many, many staff unhappy and they feel like they’re getting mixed messages” (Medical leader A).

Many changes to policy and practice occurred throughout the 16-month study period. Among the most significant changes to service provision were those relating to the procurement, education, and usage of PPE, causing confusion, outrage, and distress among staff. Information regarding correct PPE use was scarce: “we got the communication that ‘you should all be wearing masks’, there weren’t enough masks for everybody [staff] to be wearing them and changing them” (Medical leader B). There was a lack of education and no consistency across institutions. This leader detailed the challenges in not having adequate information to alleviate staff angst: “staff are asking lots of questions that at the moment we don’t have the answers for, which includes like, “should we be wearing scrubs? Where can we get scrubs? What sort of PPE should we be wearing in different circumstances? What is the guideline, what are we doing about this?” (Midwifery leader B). They also explained conflicts regarding appropriate PPE use, “we’re overusing PPE. We are not giving clear messages, we are changing our minds every day and there’s a whole debate and particularly in the maternity services space, they are multi-disciplinary team approach, the views of the multi-disciplinary team in relation to women, particularly in labour … we are not on the same page” (Midwifery leader B).

Sharing new information to staff and patients remained a constant throughout the study period, a process made easier as information updates became more rapid and systematic.

#### The need to support people

All leaders talked passionately about being committed to supporting their peers, staff and patients throughout the pandemic. One leader said, “I meet together regularly with the nursing leaders so we have those same conversations around how the team is doing, what are we doing to support the team, is there anything else we can be doing, so, there are plenty of opportunities. I find time in my team to reflect and chat with team members and get a feel for where they are up to at the moment as well” (Medical leader D). Leaders acknowledged that they were often the people that their staff members would confide in and share their stressors. Another leader reported that, “[the staff] share things with me that they wouldn’t share with a lot of other people and there’s trust there … I don’t have all of the answers, but sometimes I provide something that gives them a pathway to progress” (Midwifery leader C). Additionally, they also provided a sense of normality for staff, one participant confided that “some days I definitely have to just put the bright shiny [face] on because I think that’s what the team need from me, they also can see that I am very human and some days they can see that that absolutely takes effort … because I think that that’s been forgotten along the way, so I think that’s been a good learning for other people as well” (Unit manager B).

Many of the leaders took it upon themselves to enquire about the mental health and wellbeing of their staff and made efforts to support them during difficult times, such as speaking to staff personally and ensuring leave or breaks from work were planned, “sometimes you are looking after everybody but actually you have to make dedicated time for each person … I ask ‘how are you doing, what is it that you need, what’s going on for you?’ and I make a point about asking what is happening at home” (Medical leader A). Some organisations also provided staff with wellness hubs and check ins, “I think the organisation and the Department [of Health] have done a really good job at providing support and then realising, even with the childcare for the healthcare professionals, providing free childcare … so I think we’re quite lucky and our organisation has set up a wellness hub for staff which is open every day between 9 and 6pm and that’s just a place they can go and you know, they are offering confidential counselling, massages or just a nice quiet place to switch off, trying to give them back some break time” (Medical leader E).

One of the major challenges was maintaining staff morale as Victoria fluctuated in and out of lockdowns: “I think on top of everything else and now with another lockdown I think our biggest challenge is to keep the staff motivated, it’s really difficult” (Medical leader E). At times, social distancing measures meant that staff did not have many opportunities to come together to debrief, “… even the tea room, you were all spaced out and you couldn’t have lunch with your friends, so you couldn’t go to the café together and sit down and have a coffee and even meetings … [it was] very different and impersonal” (Unit Manager A).

Like many other non-urgent procedures and services, professional development for staff, such as clinical training, were indefinitely postponed or conducted online. One leader adapted their services by introducing “online education; so, videos of knot tying and descriptions and some sutures and then we have a shorter session to get together for them to practice, so that we can keep going on because if you … we don’t know how long this, we can’t just keep, we can’t just stop education.” (Midwifery leader A). Leaders recognised the impact stopping education would have on safety and career trajectories and advocated for these education programs to be completed in a COVID-19 safe manner. Efforts were made to develop alternative resources, one participant stated, “[I think we have] got the hang of what education looks like for the frontline workforce now because everything is in online platforms, we do very little face-to-face and if it is face-to-face it’s 15 minute sessions in full PPEs, so I think that we have really sort of refined that process now and it’s just second nature” (Unit manager B). This was also challenged by another leader, stating, “A lot has gone online but if you need to do an assessment you still need to do it face-to-face and one of the things that we have had to push for is doing the neonatal resource assessments face-to-face, yes, it has had to change a bit, it’s such a vital part, you can’t just say “oh just do it online, you can watch it.” So, we have cut down the amount of contact time, been very aware of the cleanliness, which I think is probably a good thing” (Midwifery leader A).

The leaders took it upon themselves to support midwifery and medical students, junior doctors and new medical staff. They recognised that at times over the 16 months students had limited opportunities to learn in a clinical environment hindering their ability to have hands on experience. Leaders wanted to support students, however due to density limits and physical distancing requirements, this was sometimes difficult. “I feel the most sorry for the trainees and the [new] graduates and students … this massive impact on their training and ability to get exposure to births and those sorts of things I really feel for that group” (Medical leader A).

Support for staff also meant advocating for people during times of uncertainty: “when you are in a senior position, you are less likely to be in contact with patients for a long time, … but it is the midwives, nurses, junior doctors who are on the true front line, who are providing that care … who are likely to acquire the infection and they are the ones who do not have a voice and so, I think it is really important that people like me who do have a voice should keep on talking about it … so that then that momentum builds up from lots of different places for things to happen” (Medical leader C). They also advocated for patients, their support people and families during labour and birth as well as on the postnatal ward.

#### Changes over time

Initial interviews with maternity care leaders in March and April 2020 set the scene for what was happening in maternal healthcare settings across the state. Leaders were at different stages of learning about the virus, some quickly realising that COVID-19 may have a detrimental effect early on (Midwifery Leader A, Midwifery Leader C, Unit Manager A and B, Medical Leader A) and others who had fewer initial concerns about the virus (Medical Leader B-F, Midwifery Leader B). In subsequent interviews, leaders described the different methods in which their health services were adapting to health policies and restrictions placed on the community. At times leaders reported similar interventions and strategies, whilst at other time points strategies differed and were unique to the health service. Almost everything changed over this time period and leaders were in a constant state of reflecting, responding, adapting and unravelling policies and practices.

In the beginning of our study period (March-June 2020), it was clear that many of the changes to maternity care services and health services more broadly, were rushed and haphazardly put together due to ever present uncertainty and later increasing cases in Victoria. Services needed to quickly adapt to increased community transmission, anticipating higher hospital admissions. Maternity services were scaled up and ready to manage influxes of COVID-19 positive women, but in many cases this was not necessary as the number of pregnant women with COVID-19 remained low. However, the impact of the maternity services was significant and every service continually had to adapt and change depending on the broader context on the pandemic. The over-arching theme of ‘being a maternity service leader during the pandemic was challenging’ reflected the longitudinal experiences of these leaders. There was a constant need to reflect and learn as time passed and towards the end of the study period, leaders were able to demonstrate growth in their ability to adapt and manage crisis as they came. Table 2 reflects exemplar quotes from leaders that were able to highlight the changes that occurred over time.

**Table 2:** Examples of the key changes over time evident in participant responses to the series of interviews across the 16-month study period

Leader	Quotes at different time points throughout the study	Link to theme/sub-themes
Senior executive	**Quote 1 (April 2020)**: “So the biggest stressor for the staff on the floor has been about PPE and it has come because of people’s interconnectedness, so our executive very early on took the position that what DHHS say is the rules … and as you know, this is has been pretty conservative in terms of their PPE guidance, but [some] midwives know someone at [Hospital 1], knows someone at [Hospital 2], knows someone at [Hospital 3], … they are all wearing scrubs for their whole clinic, they are all wearing a surgical mask for all their clinic … so all of the first 2 weeks of April was all about PPE. **Quote 2 (September 2020)**: “I spent all of my holidays on email, managing anxiety primarily about PPE. People wanting to wear masks, wanting to wear scrubs, wanting strict visitation, but at that time that was not the recommendation from DHHS” **Quote 3 (June 2021)**: “What went well was that not needing to individualize your institution and the rules, “these are the rules, from the department” so that was a really good thing … I think what’s different now is that that information from the Department to the hospital to the staff is more rapid and systematic than it used to be, somewhat ad hoc.”	Needing to adapt and alter services - guideline development and changes Needing to filter and translating information – PPE guidelines
Maternity unit manager	**Quote 1 (April 2020)**: “My head of unit and myself have been very consistent in our messaging to our workforce, both nursing and medical, since late February. So we send out an email three times a week to our staff base where we collate information relevant to our staff base and put it into a single email because obviously people get information from everywhere, its information overload.” **Quote 2 (June 2021)**: “it’s been about making sure that the staff have the information they need because they are being inundated and the messaging within [Hospital] is very good and we are often ahead of the messaging that comes out from the department, so it’s been about filtering that. That takes a bit of time and it took a bit of getting used to as well and figuring out the best means of communication for the team because at the end of the day communication is key, so that’s probably been the hardest thing to figure out what works well and same thing doesn’t work, like if you have got an urgent message that’s different to your weekly wrap up emails or whatever happens to be and I think there is email fatigue, I think a lot of people have email fatigue at the moment and I think that’s hard when that’s the expectation and how you going to get your information out to your staff.”	Needing to filter and translating information – communication channels, what works best
OBGYN	**Quote 1 (March 2020)**: “We are still waiting on workflows and clinical practice guidelines for staff and it’s increasing staff anxiety, so much like we’ve seen at a national level, politically, people actually want information and want transparency and I think that that would actually help a great deal in terms of staff anxiety and preparedness”. **Quote 2 (June 2021)**: “Initially we did a lot of simulation specifically to design the protocols and guidelines and everything for COVID and then donning and doffing and PPE and then as things progressed we didn’t really need to do that quite as much because people were familiar with that and then as we were coming out of lockdown, we started fit testing, so also people were getting a bit more education about PPE but also I think there was a lot more awareness of the new guidelines that had moved to actually recognition of COVID is airborne”	Needing to filter and translating information – guideline development and dissemination The need to support people – facilitating education

## Discussion

This novel longitudinal qualitative study has documented the experiences of eleven maternity service leaders during the first 16-months of the pandemic from March 2020. Leaders across Victoria shared their experiences in the provision of care during the pandemic. Key roles and responsibilities of leaders included: needing to be rapid decision makers, needing to adapt and alter services, needing to filter and translate information, as well as the need to support through the pandemic. Change was captured over time by leaders’ ability to adapt and respond accordingly to maternity care system reform under the pressures of the pandemic. Leaders had to navigate the challenges of their key roles and responsibilities and shared insightful experiences during the pandemic. Processing information quickly as new government announcements were made, advocating for clear information, and being present and available to their staff were some of the qualities described by leaders throughout the pandemic.

Our findings are consistent with other studies that have documented the impact of the COVID-19 pandemic on maternity services and healthcare staff [18, 38–41]. Across maternity services, there was a rapid transition to telehealth and remote care, infrastructure changes to enforce public health measures (i.e. social distancing), staff shortages and redeployment, as well as changes to labour care and birthing plans for women and their families [40, 42]. Commentaries published in 2020 emphasised the importance of healthcare leadership and suggested qualities that are required to deliver high quality care included: remaining calm, demonstrating clear communication, the need to coordinate and collaborate with outside partnerships, provide support and, have clear and humble leadership [43–45]. Wilson et al. 2021, describe the importance of ‘caring for the carers’ ensuring maternity provides are well supported so that they are able to provide the best quality care to women and newborns in times of uncertainty and unknown [23].

An Australian study exploring the experience of primary health care nurses highlights similar challenges to our study as well as highlighting/identifying the need for nurses to be acknowledged by management and leadership staff [46]. There were concerns regarding lack of PPE, need for information sharing and communication as well as improvements in the delivery of messages [46]. Leaders waiting for clear directives from hospital directors and government public health offices, perceived difficulty in managing the information that staff received from alternative sources, in particular whether or not PPE was required, and in what circumstances. An uncoordinated response from leaders, appeared to hinder streamline communication channels and create uncertainty and confusion in staff. Early in the pandemic, this process was fraught with difficulty as information dissemination often relied on approvals from the health department, creating a lag between policy and implementation. With time, leaders were able to successfully adapt policies ad hoc, as fewer changes needed to be made to policy documents. However, a transparent and streamlined communication channel between policy makers, maternity service leaders and their frontline staff are required to ensure that they are able to access up to date information to provide high quality care, and there is consistency in their pandemic response across the state.

Leaders frequently commended their staff’s resilience to the constant changes and demands of the pandemic, but like all healthcare staff around the world, the ongoing lockdowns and public health restrictions in 2020 and 2021, meant that they were also a tired workforce [18, 23, 47]. A UK study reported the mental health states of health care workers during June and July 2020; they reported high levels resilience in staff, as well as good organisational supports, but also detected increased burnout, depression and anxiety amongst health care workers [48]. Managing burn out and fatigue was a constant challenge for leaders, especially for the workforce in Metropolitan Melbourne where extended lockdowns were endured [13]. The leave imbalance during the lockdown periods as fewer staff took leave ultimately affected how leave was managed as restrictions eased towards the end of the study. In order to maintain and preserve a resilient workforce, opportunities to debrief, policies to encourage self-care and workforce planning including reasonable shifts and rosters [49], and this was a challenge leaders had to manage and overcome throughout the study period. Lessons learnt from previous health emergencies indicate that developing and maintaining an organisational culture of resilience may reduce the expected stress of pandemics on healthcare workers [50]. Developing a framework for maternity care staff that fosters individual resilience and includes coping mechanisms and psychological first aid, as well as organisational resilience, such as establishing reserves and supplies (e.g. medication, equipment and PPE) prior to crisis, back-up plans, and training for staff to work in unfamiliar environments as recommended by Maunders et al., and Wilson et al., [23, 51] may be the stepping stone for maternity care services.

### Strengths and limitations

This is a longitudinal qualitative study that prospectively conducted a series of interviews with leaders across multiple maternity services in Victoria, allowing the collection of real-time data, and reducing information and recall bias. Leaders of health services are infrequently studied, however, this specific cohort provided valuable insight into how maternity services function, some of the complexities as a profession, their role and responsibilities as a leader and how this changed over time. Most interviews were conducted within a few weeks or months of each other to ensure that a clear understanding of the events that occurred over the study period were able to be documented with a high attention to detail. The large number of interviews contributed to an extensive dataset providing rich responses and allowed ample data for analysis. In addition, the research team conducted multiple rounds of theme and sub-theme development, using these opportunities to de-brief and acknowledge authors’ own preconceptions and to ensure analysis and coding of data was unbiased.

Leaders were purposively sampled across Victoria, from multiple health services in various leadership and management roles. One limitation is that only Victorian maternity care leaders were interviewed, therefore a national perspective from leaders within maternity services was not gained. However, during the study period – Victoria was the state most affected by the COVID-19 pandemic in terms of case numbers but also public health measures to prevent transmission. Another limitation was that we excluded leaders from level 1–3 maternity services (local care for low-risk mothers and babies), missing the opportunity to explore their experiences, how they managed their own challenges with staff shortages and acutely sick COVID-19 positive women. It is likely that the non-inclusion of these leaders would not significantly impact the findings as most level 1–3 maternity services are from regional and rural areas of Victoria, which experienced relatively low levels of community transmission, compared with metropolitan Melbourne. One leader from a tertiary hospital servicing some of these areas was included in the study to provide some perspective into the regional response to the pandemic, but it must be acknowledged that the findings are largely based on the metropolitan experience.

### Implications for health policy and practice

The COVID-19 pandemic continues to demand a highly flexible and adaptable maternity care sector to implement policy and practice changes within the working environment. It has provided a significant number of challenges and it is evident that services need to be better prepared for future rapid changes to care. Specifically, the interviews with maternity leaders indicate that: a reserve of PPE is required to ensure the safety of all staff, at all times; guideline development and dissemination should involve a multidisciplinary approach; and better communication channels between state level health departments and individual facilities are required to ensure a unified pandemic response for maternity care is shared across the state.

The implications of the study findings highlight the invaluable experiences, expertise and knowledge that leaders have gained during this pandemic. These findings will be important in similar future crises. COVID-19 has also shone a light on the importance of treating maternity services as key essential services, and ensuring they are well integrated into the wider health system. Maternity services are unique in that women will continue to give birth during emergencies, and to cross into intensive and critical care services that cannot be put on hold, unlike elective surgery. The findings highlight how systems could be strengthened, especially communication channels between state level health departments and with individual facilities and how they in turn communicate with other health services, and their staff.

## Conclusion

Maternity care leaders played a played an essential role in responding to the huge burden and challenges brought on by the COVID1-9 pandemic. They offer invaluable insights that would improve the design of a response system. It is crucial that we learn from their experiences so that high quality care can be provided for childbearing women and their families in future crises.

## Electronic supplementary material

Below is the link to the electronic supplementary material.

Additional file 1: Appendix 1

Additional file 2: Appendix 2

## Data Availability

The datasets generated and/or analysed during the current study are not publicly available due to the sensitive nature of the information captured during the interviews but are available from the corresponding author on reasonable request.

## List of abbreviations

LQR: Longitudinal qualitative research
COREQ: Consolidated criteria for reporting qualitative studies
DHHS: Department of Health and Human Services
SCV: Safer Care Victoria
CCOPPM: Council on Obstetric and Paediatric Mortality and Morbidity
NICU: Neonatal intensive care unit
RANZCOG: Royal Australian and New Zealand College of Obstetricians and Gynaecologists
RCOG: Royal College of Obstetricians and Gynaecologists
GP: General practitioner
OBGYN: Obstetrician gynaecologist
