# Supplementary material for: Challenges of being a maternity service leader during the COVID-19 pandemic: a descriptive analysis of the journey
Source: BMC Pregnancy Childbirth. 2023 Apr 24;23:279. doi: 10.1186/s12884-023-05614-5 (PMC10123468; doi:10.1186/s12884-023-05614-5)
Supplement: Supplementary file 2 — Additional file 2: Appendix 2 [file 12884_2023_5614_MOESM2_ESM.docx]

**Appendix 2: Participant Information Sheet**


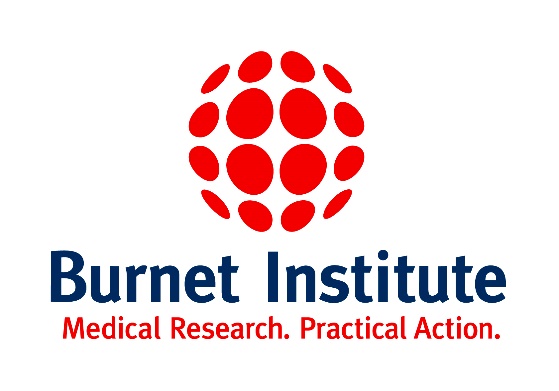


***Participant Information Sheet***

**COVID19 Maternity Services Planning and Impact: The COVID19 Journey Project**

WHO IS DOING THE RESEARCH?

Professor Caroline Homer, Dr Alyce Wilson and Associate Professor Joshua Vogel are leading this research. We are a midwife and two public health doctors with extensive experience in maternal health research. We are based at the Burnet Institute in Melbourne.

WHAT IS THIS RESEARCH ABOUT?

The purpose of project is to document the maternity health system response to the COVID19 pandemic, especially the planning, processes and subsequent impact on the health workforce and on the women and babies. The findings will provide critical insights to guide maternity service preparedness for future pandemics or emergency situations.

IF I SAY YES, WHAT WILL IT INVOLVE?

We will invite you to participate in a series of brief interview that will take less than 20 minutes each at a time that is convenient for you. The interviews will be conducted 1-4 weeks apart over a 6-month period as the COVID19 pandemic unfolds in Victoria. The interviews will use the online platform Zoom.

With your consent the interview will be digitally voice recorded to allow accurate transcription of your responses. Any information that is obtained in relation to this study and that can be identified with you will remain confidential.

If you provide permission by signing the consent form, you will have the opportunity to review the transcripts and also the findings and interpretation. Together with the research team you will have an opportunity to decide how the findings should be presented.

Your audio-recording will be transcribed by a transcription company/service who will be required to sign a confidentiality agreement prior to being provided with the files using a secure cloud-based system. We will have all the interviews transcribed by the end of the project (November-December 2020).

The electronic recordings will be stored in a secure password-protected cloud storage file whilst they are being transcribed and then destroyed. All transcribed data will be stored securely and will be deleted within 5 years of completion of the study. Only the three investigators will ever have access to the transcripts.

ARE THERE ANY RISKS/INCONVENIENCE?

It is possible that you may become distressed telling your experience. The interviewers (Professor Homer and Dr Wilson) will be acutely aware of this possibility and will ensure that all interviews are conducted with kindness, empathy and compassion. Access to support, such as the health system’s Employee Access program will be made available (this support is free of charge). There are also some great resources on the Beyond Blue website

https://www.beyondblue.org.au/

The researchers will ‘check back in’ with you after each interview (using email or text message) to ensure that you are OK given the circumstances. If at any time you feel that continuing with the interview or future interviews is too distressing, we will halt the interview.

We also know that you may not always be able to participate in a scheduled interview due to clinical demands or if you yourself becomes unwell. There will be a high degree of flexibility in continuing with the scheduled interviews and participants will be to cancel and/or reschedule.

It is possible that you could be potentially identified from the audio recordings (i.e. through your voice). Prior to transcription, the recordings will be kept in a password protected file accessible only by Caroline Homer and Alyce Wilson. We will transcribe the recordings as soon as possible and then we will destroy the recording to minimize this risk.

Sometimes, raw data is made available to others in a de-identified form. We do not plan to do this due to the sensitive nature of the raw data. There is no obligation to do so by a funder and we feel that not doing so is more protective of your privacy.

WHY HAVE I BEEN ASKED?

You have been identified as a potential participant because you are working as a clinician (obstetrician, midwife, neonatologist or obstetric anaesthetist) or in a senior policy position, in Victoria.

DO I HAVE TO SAY YES?

You don’t have to say yes.

WHAT WILL HAPPEN IF I SAY NO?

Nothing. I will thank you for your time so far and won’t contact you about this project again.

IF I SAY YES, CAN I CHANGE MY MIND LATER?

You can change your mind at any time and you don’t have to say why. I will thank you for your time so far and won’t contact you about this research again. All your data (recordings and any transcribed data or notes) will be destroyed and will not be used.

WHAT IF I HAVE CONCERNS OR A COMPLAINT?

If you have concerns about the research, please feel free to contact me on Caroline.homer@burnet.edu.au or 0418 466 974.

If you have any complaints about any aspect of the project, the way it is being conducted or any questions about being a research participant in general, then you may contact:

HREC Office/Complaints contact person

Position: Complaints Officer, Office of Ethics & Research Governance,

Alfred Health Telephone: (03) 9076 3619

Email: [research@alfred.org.au](mailto:research@alfred.org.au)

Please quote the following Project ID number: 63017”

**Appendix 2: Consent form**


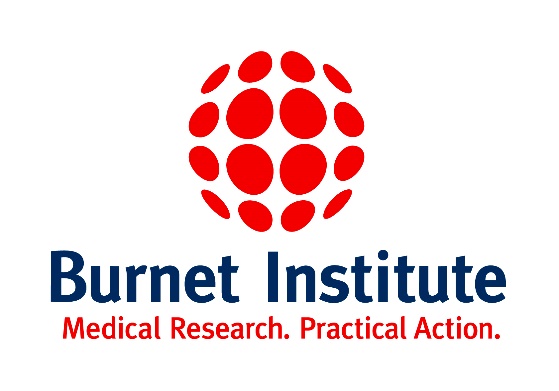


**COVID19 Maternity Services Planning and Impact: The COVID19 Journey Project**

Name of Participant: ______________________________________________________________

1. I consent to participate in this project, the details of which have been explained to me, and I have been provided with a written plain language statement to keep.
2. I understand that the purpose of project is to document the maternity health system response to the COVID19 pandemic, especially the planning, processes and subsequent impact on the health workforce and on the women and babies. The findings will provide critical insights to guide maternity service preparedness for future pandemics or emergency situations.
3. I understand that my participation in this project is for research purposes only.
4. I acknowledge that the possible effects of participating in this research project have been explained to my satisfaction.
5. In this project I will be required to participate in a series of in-depth semi structured interview based on some pre-arranged questions, conducted by a member of the research team.
6. I understand that my interviews will be audio recorded and transcribed.
7. I understand that my participation is voluntary and that I am free to withdraw from this project anytime without explanation or prejudice and to withdraw any unprocessed data that I have provided.
8. I understand that the data from this research will be stored at the Burnet Institute and will be destroyed after 5 years. The recording of my voice will be destroyed as soon as my interview is transcribed so that I cannot be recognised.
9. I have been informed that the confidentiality of the information I provide will be safeguarded subject to any legal requirements; my data will be password protected and accessible only by the named researchers.
10. I understand that after I sign and return this consent form, it will be retained by the researcher.
11. I understand that at the end of the project (end 2020), I will be given a copy of my transcripts and I will have the opportunity to review or edit my responses if I feel they were incorrectly transcribed.
12. I understand that I will have the opportunity to review the findings and interpretation and together with the research team a decision as to how the findings should be presented will be made.

Participant Signature: ________________________________ Date: _______________________

Investigator Signature: _______________________________ Date: ________________________
